# Tyrosine Kinase Inhibitors Induce Down-Regulation of c-Kit by Targeting the ATP Pocket

**DOI:** 10.1371/journal.pone.0060961

**Published:** 2013-04-23

**Authors:** Diane D'allard, Julie Gay, Clotilde Descarpentries, Emilie Frisan, Kevin Adam, Frederique Verdier, Célia Floquet, Patrice Dubreuil, Catherine Lacombe, Michaela Fontenay, Patrick Mayeux, Olivier Kosmider

**Affiliations:** 1 Institut Cochin, Département d'Immunologie-Hématologie, Paris, France; 2 INSERM U1016, Paris, France; 3 Centre National de la Recherche Scientifique, Unité Mixte de Recherche 8104, Paris, France; 4 Université Paris Descartes, Faculté de Médecine, Paris, France; 5 Equipe Labellisée Ligue Contre le Cancer, Paris, France; 6 LABEX (Laboratoire d'Excellence) GR-Ex, Paris, France; 7 INSERM, U1068, CRCM, Centre de Référence des Mastocytoses-CEREMAST; Institut Paoli-Calmettes, Marseille; Aix-Marseille Université; CNRS, UMR7258, Marseille, France; 8 Assistance Publique-Hôpitaux de Paris, Hôpital Broca-Cochin-Hôtel-Dieu, Service d'Hématologie Biologique, Paris, France; 9 Proteomic Platform of the Paris Descartes University (3P5), Paris, France; Institut national de la santé et de la recherche médicale (INSERM), France

## Abstract

The stem cell factor receptor (SCF) c-Kit plays a pivotal role in regulating cell proliferation and survival in many cell types. In particular, c-Kit is required for early amplification of erythroid progenitors, while it must disappear from cell surface for the cell entering the final steps of maturation in an erythropoietin-dependent manner. We initially observed that imatinib (IM), an inhibitor targeting the tyrosine kinase activity of c-Kit concomitantly down-regulated the expression of c-Kit and accelerated the Epo-driven differentiation of erythroblasts in the absence of SCF. We investigated the mechanism by which IM or related masitinib (MA) induce c-Kit down-regulation in the human UT-7/Epo cell line. We found that the down-regulation of c-Kit in the presence of IM or MA was inhibited by a pre-incubation with methyl-β-cyclodextrin suggesting that c-Kit was internalized in the absence of ligand. By contrast to SCF, the internalization induced by TKI was independent of the E3 ubiquitin ligase c-Cbl. Furthermore, c-Kit was degraded through lysosomal, but not proteasomal pathway. In pulse-chase experiments, IM did not modulate c-Kit synthesis or maturation. Analysis of phosphotyrosine peptides in UT-7/Epo cells treated or not with IM show that IM did not modify overall tyrosine phosphorylation in these cells. Furthermore, we showed that a T670I mutation preventing the full access of IM to the ATP binding pocket, did not allow the internalization process in the presence of IM. Altogether these data show that TKI-induced internalization of c-Kit is linked to a modification of the integrity of ATP binding pocket.

## Introduction

The stem cell factor (SCF) receptor c-Kit (also referred as CD117) regulates cell survival, proliferation, and differentiation. C-Kit is a member of the type III subfamily of receptor tyrosine kinase (RTK) that also includes the receptors for M-CSF, Flt-3, and PDGF. Physiologically, c-Kit is expressed on melanocytes, germ cells, mast cells and hematopoietic progenitor cells. C-Kit is required for early erythroid progenitor amplification while its expression must be down-regulated for cells entering terminal differentiation. Accordingly, mice with mutations in the W or Sl Locus encoding c-Kit or SCF respectively, present with a strong anemia [1].

The recent description of the crystal structure of the entire ectodomain of c-Kit before and after SCF stimulation helps the comprehension of c-Kit biology [2]. Indeed, the main consequence of SCF binding is to bring together two molecules of c-Kit. After ligand binding, c-Kit is phosphorylated and rapidly internalized. However, the fact that intrinsic tyrosine kinase activity is required for driving the internalization of a receptor is still controversial [3]–[6]. Expression at plasma membrane and ligand-mediated internalization of active mutants of the kinase domain vary according to the targeted residue and, for a given residue, to the type of substitution [7]. For instance, mutation of c-Kit autophosphorylation Y821 or substitution of D816 by a valine or a tyrosine does not abrogate ligand-induced receptor internalization [7, and personal data], while the G559D c-Kit mutant is stabilized at the plasma membrane in the presence of SCF [8]. However, it has been shown that kinase dead mutant of c-Kit is still able to internalize in response to ligand binding, although the rate of internalization decreases. This is consistent with the internalization of the epidermal growth factor (EGF) receptor to occur even if the receptor is inactive [5]. This suggests that ligand binding or ligand-induced dimerization could be the sole determinant for RTK internalization, independently of tyrosine kinase activation.

Activated c-Kit is then targeted for endocytosis and degradation by the lysosomes. This step requires the ubiquitin ligase Cbl that associates with the tyrosine-phosphorylated receptor. The recruitment of Cbl to c-Kit involves both the C-terminal part of the receptor and its membrane proximal domain. It has been shown that isoleucine 787 is implicated in the internalization process of c-Kit in mice [3]. A substitution of isoleucine by phenylalanine (I787F) which does not affect SCF binding, strongly impairs c-Kit internalization due to ineffective activation of Cbl. The transmembrane domain also recruits Src family kinases that have been shown to participate to Cbl-dependent ubiquitination of c-Kit [9]. Inactivating mutations of *CBL* gene responsible for wild type (wt) c-Kit overexpression have been identified in myeloproliferative disorders or mastocytosis [10]. Furthermore, expression of an activated mutant of c-Kit, or a deregulated production of SCF have been implicated in the pathophysiology of leukemias, mastocytosis, gastrointestinal stromal tumors and lung carcinomas for a long time [for review, 11]. Therefore, c-Kit may represent an attractive target for many therapeutic approaches.

Tyrosine kinase inhibitors (TKI) like imatinib (IM) or masitinib (MA), which enter the ATP binding pocket and competitively inhibit ATP binding and receptor kinase activity, have been shown to block constitutively active mutants [12]–[16]. The clinical response to these compounds depends on the type of mutations. Furthermore, the knock-down of wt c-Kit overexpression, using a siRNA strategy efficiently reduces c-Kit-driven cell proliferation [16]. Thus, either direct targeting of active mutant or inhibition of c-Kit expression are two different ways for reducing c-Kit tumorigenicity.

In the present work, we report that down-regulation of wt c-Kit is efficiently driven by IM or MA that induce c-Kit internalization and its subsequent lysosomal degradation in the absence of ligand. Accessibility of IM to the ATP binding pocket of c-Kit is required for the internalization process to occur. In normal erythroblasts, c-Kit down-regulation by IM forces Epo-dependent final maturation.

## Materials and Methods

### Reagents

Highly purified recombinant human SCF was from Miltenyi Biotech (Bergisch Glabach, Germany) and Epo was a gift of Dr M. Brandt (Roche, Penzberg, Germany). Insulin-like growth factor (IGF)-1 and dexamethasone were purchased from Sigma Aldrich (St Louis, MO). Imatinib mesylate (IM) and masitinib mesylate (MA) come from the laboratory of Dr P. Dubreuil. The profteasome inhibitor N-Ac-Leu-Leu-norLeucinal (LLnL) was purchased by Calbiochem (Merck Biosciences, Darmstadt, Germany). Methyl-β-cyclodextrine (MβCD), methylamine and cycloheximide (CHX) were purchased from Sigma Aldrich. Sorafenib was obtained from Santa Cruz (SantaCruz Biotechnologies, SantaCruz, CA). For flow cytometry, CD117 antibodies to c-Kit coupled to phycoerythrin cyanin 5 (PC5) was purchased from Beckman Coulter (Miami, FL). Antibodies directed against c-Kit for immunoblot and immunoprecipitation were purchased from Cell Signaling (Cat ref. 3074, Danvers, MA), anti-Kit phosphotyrosine (pKit-Y719) were from Cell Signaling (ref. 3391) anti-Epo-R antibodies were from SantaCruz, and anti-actin antibodies were from Sigma Aldrich.

### Cell lines and culture conditions

Human UT-7/Epo cells previously described by Komatsu and colleagues [36] and were cultivated in α-minimum essential medium (αMEM) containing 10% fetal calf serum, 1 mM glutamine, 100 UI/ml penicillin-streptomycin and 1 U/ml Epo. UT-7/Epo cells were stably transfected by electroporation with pcDNA1 plasmid encoding hemagglutinin (HA)-tagged wt c-Cbl or the 70Z c-Cbl mutant that lacks E3 ligase activity, and transfected cells were selected using G418. Plasmids encoding *wt* c-Cbl and 70Z c-Cbl were a gift from Pr Y Yarden from the Department of Biological Regulation, at Weizmann Institute of Science (Rehovot, Israel). For SILAC experiment, UT-7/Epo cells were cultivated in MEM medium minus L-Lysine and L-Arginine (Pierce) supplemented with 0.1 mg/ml 13C6 L- Arginine-HCl (Pierce) and 0.1 mg/ml 13C6, 15N2 L-Lysine-2HCl (Pierce, “heavy medium”), or the same concentrations of L-Arginine (Sigma) and L-Lysine (Sigma, “light medium”) and containing 10% dialyzed fetal calf serum and 1 U/mL Epo. For a total integration of amino acids, cultures last 5 weeks. Murine BaF-3 cells previously described by Collins and colleagues [38] were transfected with a hKit WT or the gatekeeper hKit T670I mutant were cultivated under murine IL3 stimulation in Roswell Park Memorial Institute medium (RPMI), containing 10% fetal calf serum, 1 mM glutamine, and 100 UI/ml penicillin-streptomycin as previously described [37]. Before experiments, cells were selected by two days of culture without IL3 plus SCF 100 ng/mL to amplify only cells expressing an ectopic c-Kit. These cell lines were a gift from Dr P Dubreuil from the Centre de Recherche en Cancérologie, at Marseille, France. For human primary erythroblast cultures, CD34^+^ progenitors were purified on MidiMacs system (Miltenyi Biotech, Bergisch Gladbach, Germany) in the mononuclear cell fraction isolated from cytapheresis on Ficoll gradient as previously described [17]. During the amplification phase, CD34^+^ progenitors were seeded for 10 days in the presence of 1 UI/ml Epo, 100 ng/ml SCF, 40 ng/ml IGF-1 and 1 µM dexamethasone and further cultured for 7 days with 1 UI/ml Epo and 40 ng/ml IGF-1 for cells to enter the terminal erythroid maturation, as previously described [17].

### Cell stimulation

UT-7/Epo cells cultivated under Epo stimulation were treated by imatinib (IM) or masitinib (MA) or vehicle at different time points and at different concentrations. In other experiments, UT-7/Epo were preincubated with 50 µM MβCD for 30 min, 50 µM LLnL for 20 min, or 100 µM methylamine for 20 min before treatment with imatinib or masitinib or vehicle for 4 h. Alternatively, UT-7/Epo (cultivated under Epo) or BaF-3 cell lines (cultured under IL3) were treated for 4 h with 1 or 5 µM sorafenib.

### Flow cytometry

C-Kit or CD71 cell surface expression was analyzed by flow cytometry using CD117-PC-5 or CD71-FITC antibodies. All data were acquired on a Cytomics FC500 flow cytometer and analyzed using Cytomics RXP Analysis (Beckman Coulter, Inc.).

### Peptides purification and immunoprecipitation of phospho-Tyr-peptides

UT-7/Epo cells cultivated in heavy medium were treated 4 h with 2 µM imatinib while control cells cultivated in light medium were treated with DMSO alone. After two washes in PBS, 50.10^6^ UT-7/Epo cells, of each culture conditions, were resuspended in 2.5 mL of lysis buffer (20 mM hydroxyethyl piperazineethanesulfonic pH 8, 8M urea, 1 mM sodium orthovanadate, 2.5 mM sodium pyrophosphate and 1 mM sodium β-glycerophosphate), sonicated twice 30 seconds and keep in ice for 20 minutes. Lysates were centrifuged at 20.000 g for 20 minutes at 4°C. Supernatants were successively treated with DTT to reduce disulfide bonds and chloroacetamide for alkylation. Equal amounts of proteins from both samples were mixed and digested by trypsin over night at 30°C. Peptides were purified on Sep-Pack C18 Classic micro column (Waters, ref. WAT051910) and on HyperSep Hypercarb SPE Columns (ThermoScientific, ref. 60106–301). After lyophilization, peptides were solubilized in Immunoaffinity Purification (IAP) buffer (50 mM 3-(N-Morpholino) propanosulfonic acid, 10 mM sodium phosphate, 50 mM NaCl, ph 7.4) and immunoprecipitated with anti-phospho-Tyr antibodies (Cell Signaling, ref. 7902) Immunoprecipitated phospho-peptides were eluted in 1% trifluoroacetic acid and analyzed by mass spectrometry using an Ultimate 3000 Rapid Separation Liquid Chromatographic (RSLC) system hyphenated to a hybrid LTQ-OrbitrapVelos mass spectrometer (Thermo Fisher Scientific). Peptides were separated on a C18 reverse phase resin (2 µm particle size,100 A pore size, 75 µm i.d.,15 cm length) with a 27 min gradient from 100% A (2% ACN, 0.1% formic acid and 98% H2O) to 30% B (80% ACN, 0.085% formic acid and 20% H2O). The mass spectrometer was operated in a data dependent scan with a full MS scan acquired with the Orbitrap followed by up to 10 LTQ MS/MS CID or ETD spectra on the most abundant ions detected in the MS scan. ETD fragmentation was performed if one of the following events was met: z = 3 and m/z<650 m/z, z = 4 and m/z<900 m/z or z = 5 and m/z<950 m/z. Whenever neutral loss at 24.5, 32.6, 49 or 98 m/z was detected, multistage activation (MSA) was performed. Mass spectrometer settings were: full MS (AGC: 1*10^6^, resolution: 6*10^4^, m/z range 400–2000, maximum ion injection time: 500 ms); MS/MS (AGC: 5*10^3^, maximum injection time: 200 ms, minimum signal threshold: 500, isolation width: 2Da, dynamic exclusion time setting: 30 s). The fragmentation wasn't permitted for precursor with z = 1. Data were analyzed using the MaxQuant 1.3.0.5 software [18]. Mass spectrometry analyses were performed by the proteomic facility of the Paris Descartes University (3P5).

### C-Kit internalization studies

SCF or Epo was labeled using IODO-GEN (Pierce, Rockford, IL) and [^125^I]SCF or [^125^I]Epo binding was performed as previously described [19], [20]. 2×10^6^ UT-7/Epo cells were used in each assay. Saturating [^125^I]SCF (200 ng/ml) or [^125^I]Epo concentrations (1 UI/ml) were added to suppress the effects of putative modifications of receptor affinity induced by the different inhibitors used. Non-specific binding, determined using a 250-fold excess of unlabeled cytokine, was less than 5% of total binding in each case. After 2 washes in PBS pH 7.4, cell-bound radioactivity was counted. All reported data represent specific binding. Each experiment was performed at least 3 times with similar results.

### Western blotting

Cells (5.10^5^) were solubilized in 10 mM Tris-HCl pH 7.5, 5 mM ethylene diamine tetracetic acid (EDTA), 150 mM NaCl, 10% glycerol, 1% Nonidet-P40 plus protease inhibitors (Complete, Roche). After sodium dodecylsulphate-polyacrylamide gel electrophoresis (SDS-PAGE), immunoblots were performed using primary antibodies followed by secondary antibodies conjugated to horseradish peroxidase (Cell Signaling) and revealed by chemiluminescence detection (GE Healthcare). The images were captured using a CCD camera (LAS3000 from FujiFilm) and quantified using Multigauge software from Fujifilm.

### Metabolic labeling and c-Kit immunoprecipitation

UT-7/Epo cells were pre-incubated for 4 h with or without imatinib in Met- and Cys-deficient α-MEM culture medium (Sigma Aldrich, M2289) containing 5% dialyzed fetal bovine serum, 1 mM glutamine, 100 UI/ml penicillin-streptomycin and 2 U/ml Epo. After pre-incubation, the cells were pulse-labeled for 15 min in the same medium containing 0.25 mCi/ml of a mixture of [^35^S]Met and [^35^S]Cys (Translabel, Perkin Elmer, Courtaboeuf, France) and chased by incubation in a medium containing unlabeled Met and Cys. Cells were either solubilized in 150 µL of lysis buffer (150 mM NaCl, 10 mM Tris pH 7.4, 1% NP-40, 0.5% deoxycholic acid and 0.1% SDS, protease inhibitors and 1 mM sodium orthovanadate, 20 mM sodium fluorure, 1 mM sodium pyrophosphate, 25 mM sodium beta-glycerophosphate as phosphatase inhibitors). Lysates were centrifuged, cleared using unspecific antibodies, and precipitated using anti-c-Kit antibody or control immunoglobulins. Immunoprecipitates were separated by SDS-PAGE, and labeled proteins were detected by fluorography using Amplify on a Typhoon fluorescence scanner (GE Healthcare).

## Results

### Imatinib or masitinib decreases cell surface expression of mature c-Kit in human erythroblasts and UT-7/Epo cell line

**Figure 1:**
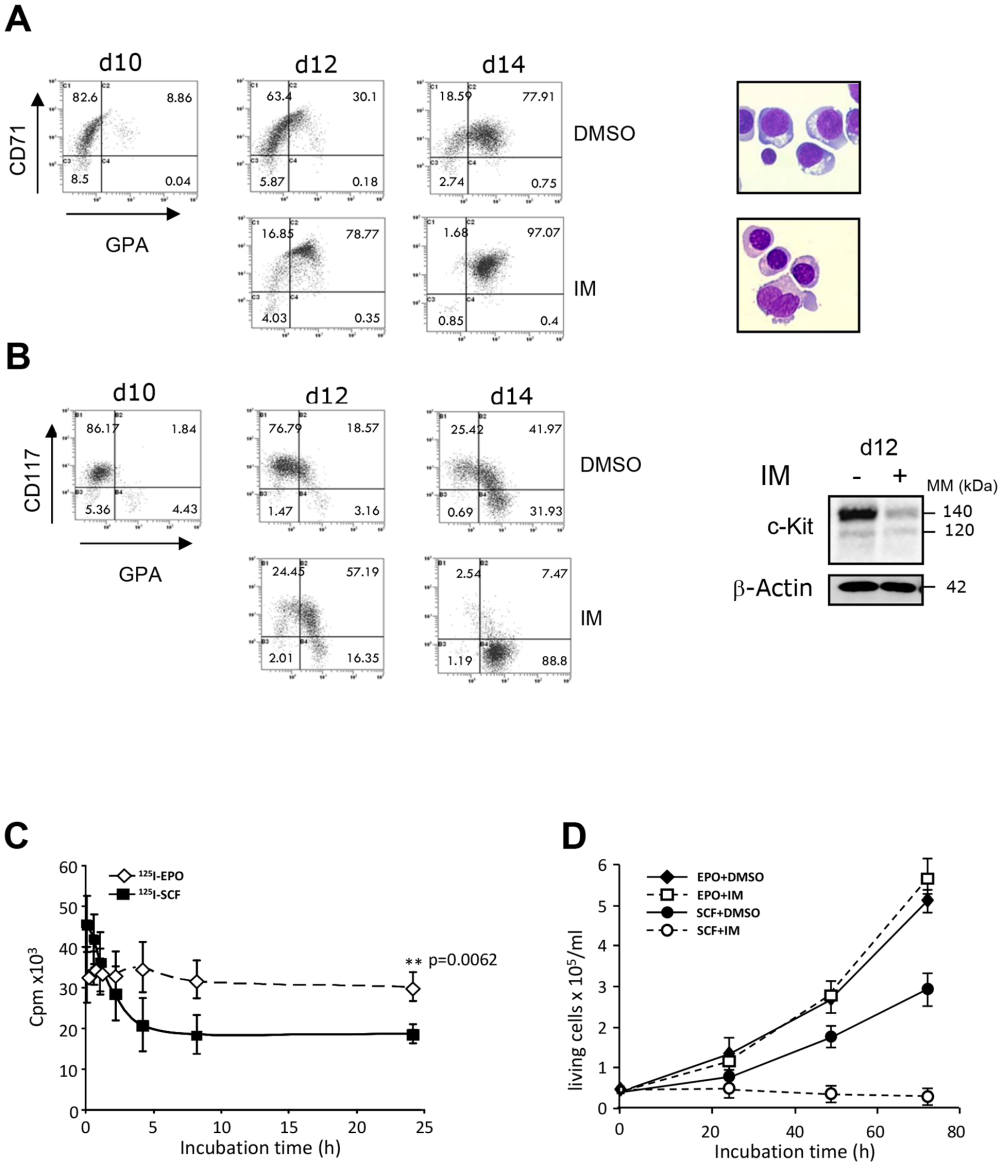
Imatinib induces the differentiation of human normal erythroblasts and decreases cell surface expression of c-Kit. Imatinib (2 µM) was added for 4 days to a culture of CD34+ cells-deriving erythroblasts. **A. Effect of imatinib on erythroblast differentiation.** Left panel: Flow cytometry for CD71 and GPA markers. Right panel: May Grunewald Giemsa staining at Day 14 (d14).** B. Effect of imatinib on c-Kit expression** Left panel: Flow cytometry for CD117 and GPA. Right panel: Immunoblot for c-Kit. **C. Expression of functional c-Kit at cell surface.** UT-7/Epo cells were incubated with 2 µM imatinib, or vehicle at 37°C for indicated times, and labeled with 200 ng/ml [^125^I]SCF or 1 UI/ml [^125^I]Epo for 90 min at 4°C. Cell surface-associated radioactivity was counted. Results are the mean of at least three independent experiments. **D. UT-7/Epo cell proliferation in response to SCF.** UT-7/Epo cells were incubated with 1 UI/ml Epo or 50 ng/ml SCF, in the presence or absence (vehicle) of 2 µM imatinib (IM). At the indicated times, cells were counted in triplicates.

C-Kit expression must be turned off for the erythroid progenitors to enter the final steps of differentiation. Here, we speculated that inhibition of c-Kit tyrosine kinase activity by TKI could facilitate erythroid cell maturation. To evaluate the effect of imatinib on erythroid cell differentiation, we used a two-phase model of *in vitro* differentiation. Using mobilized peripheral blood CD34^+^ progenitors, we obtained erythroid progenitors after 10 days of culture in the presence of Epo, SCF, IGF-1 and dexamethasone. At day 10, cells were washed and reseeded in differentiation medium containing Epo and IGF-1 either in the presence or absence of imatinib for 4 days. As shown in Figure 1A, the presence of imatinib strongly accelerated the acquisition of erythroid marker glycophorin A (GPA) after 2 and 4 days of treatment. Then we analyzed the expression of c-Kit at the time of the onset of erythroid maturation. As shown by flow cytometry, the expression of c-Kit at plasma membrane was fully inhibited after 4 days (Figure 1B, left). By Western blot (Figure 1B, right), we observed a dramatic reduction of the expression of the mature 140 kDa form of the protein.

To investigate the mechanism of c-Kit down-regulation, we used the Epo-dependent UT-7/Epo cell line that expresses endogenous c-Kit. We first assessed the specificity of imatinib on the expression functional c-Kit receptors, we quantified the specific binding of [^125^I]SCF or [^125^I]Epo to their receptors at cell surface in the presence of 2 µM imatinib incubated for 0 to 24 h at 37°C (Figure 1C). We observed that imatinib efficiently reduced [^125^I]SCF binding but did not modify [^125^I]Epo binding. Thus, imatinib reduced the level of functional c-Kit receptors, while it did not affect the cell surface expression of the Epo-R. The decrease of SCF binding was detectable after 2 h of incubation with imatinib and was maximal after 4 h. As expected, since imatinib targeted c-Kit, the UT-7/Epo cell line proliferation in response to SCF was completely abolished by imatinib. By contrast, Epo-induced proliferation was not modified (Figure 1D). These results show that imatinib induces the down-regulation of functional c-Kit at plasma membrane.

**Figure 2:**
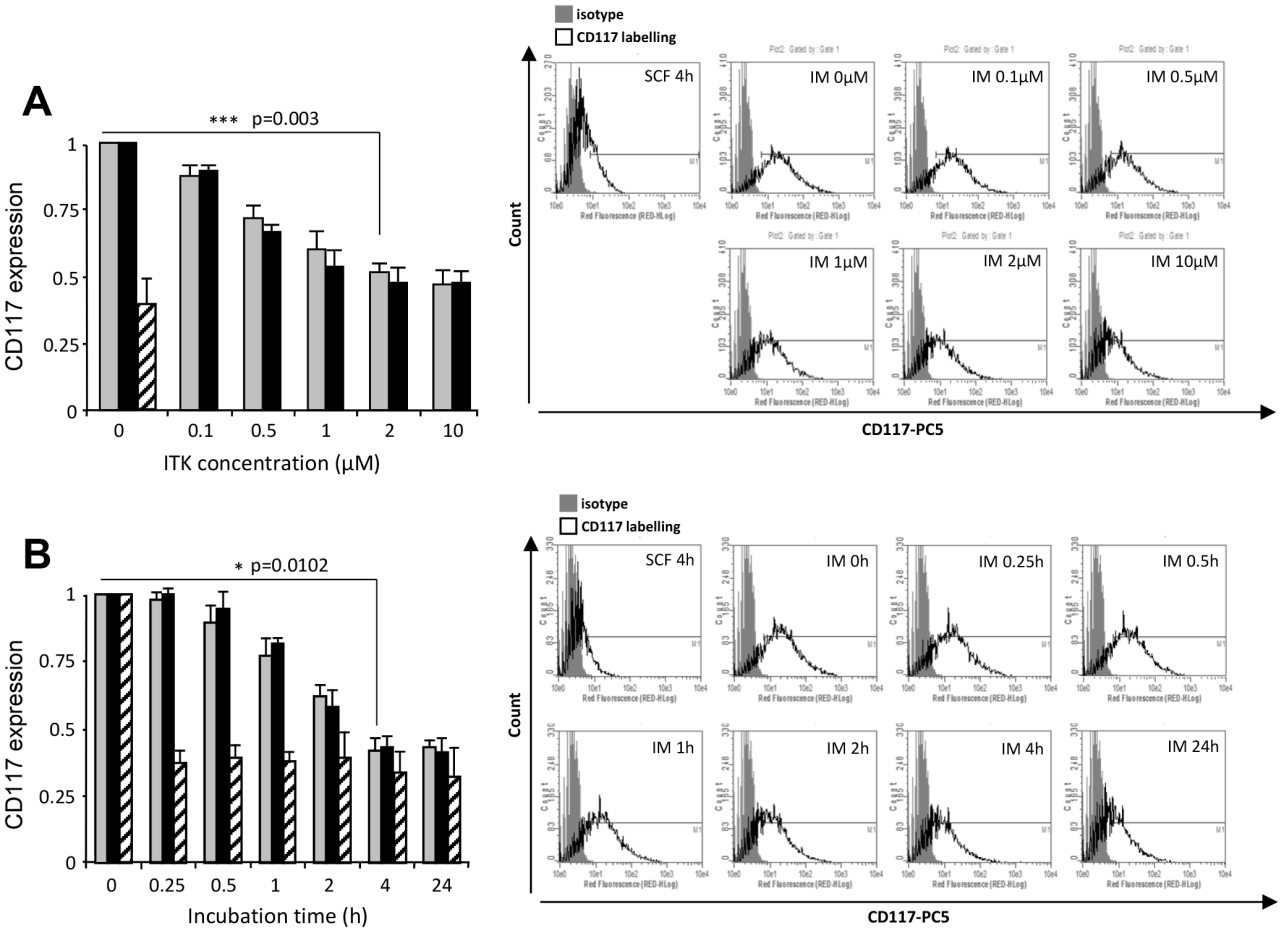
Imatinib or masitinib decreases cell surface expression of mature c-Kit in UT-7/Epo cell line. **A. Dose-dependent inhibition of c-Kit expression by TKI.** Cell surface expression of c-Kit. UT-7/Epo cells were incubated with SCF 50 ng/ml for 10 min (hatched bars) or indicated concentrations of imatinib (IM) (grey bars) or masitinib (MA) (black bars) for 4 h. **B. Kinetic of c-Kit down-regulation by TKI.** UT-7/Epo cells were incubated with 2 µM IM (grey bars) or MA (black bars) or 50 ng/ml SCF (hatched bars) for indicated times. **C-Kit cell surface expression was determined by flow cytometry.** Results were expressed as ratios of median fluorescence intensity (RFI), and the values were normalized to untreated cells. Results are the mean of at least three independent experiments.

To investigate whether the mechanism of down-regulation induced by TKI differed or not from that induced by the natural ligand of c-Kit, we compared the membrane expression of CD117 by flow cytometry after incubation with SCF for 10 min or increasing concentrations of imatinib or masitinib for 4 h (Figure 2A). In these conditions, CD117 signal expressed as a ratio of median fluorescence intensity (RFI) decreased according to the increase of inhibitor concentration. The effect was identical to that of SCF with 2 µM of each inhibitor. On the same experiments neither imatinib nor masitinib affected the expression of surface marker CD71 corresponding to the type 1 transferrin receptor (data not shown). When compared to SCF, TKI were able to induce c-Kit down-regulation with different kinetics. After 10 minutes of stimulation by SCF, cells expressed less than 50% of the initial level of c-Kit at the plasma membrane, while this maximal down-regulation was observed after 4 h of imatinib or masitinib (Figure 2B). Down-regulation remained stable after 24 h both in the presence of inhibitors or ligand (Figure 2B). We obtained the same results in human erythroid TF-1 cell line and in murine BaF3 cell line stably transfected with human *wt* form of c-Kit (Figure S1). This suggests that TKI induced a down-regulation of c-Kit through a mechanism that could differ from the down-regulation following SCF binding.

### Imatinib prevents c-Kit re-expression after ligand-induced down-regulation

**Figure 3:**
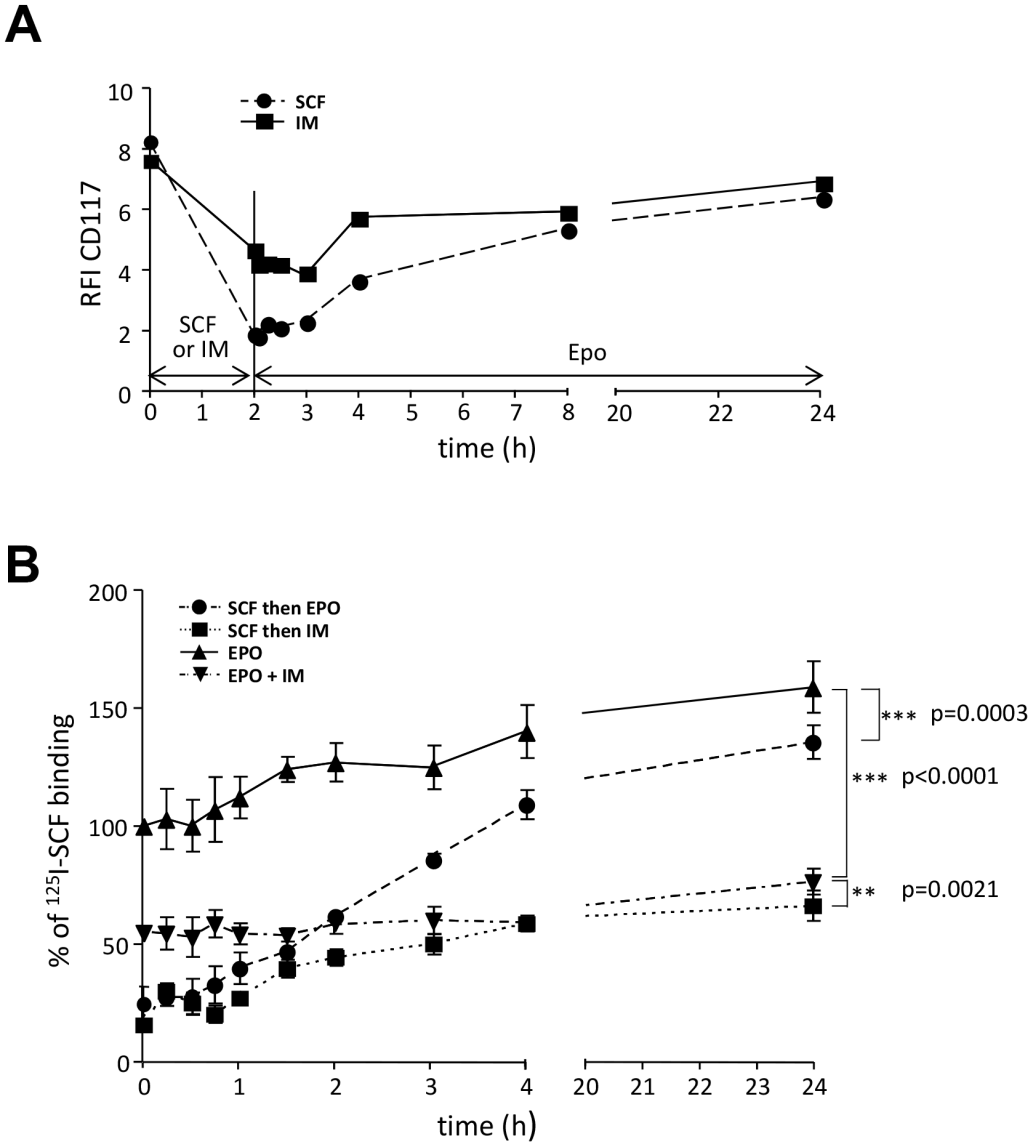
Imatinib prevents c-Kit receptor re-expression after SCF-induced down-regulation. **A. Down-regulation of c-Kit by imatinib is reversible.** UT-7/Epo cells were incubated with 50 ng/ml SCF (dotted line) or with 2 µM imatinib (filled line) for 2 h. After washing in PBS, cells were cultured with 1 UI/ml Epo for 24 h. CD117 expression was measured at the indicated times by flow cytometry and results were expressed as RFI. **B. Imatinib prevents the re-expression of functional c-Kit after SCF binding.** UT-7/Epo cells were pre-incubated with 50 ng/ml SCF. After washing in PBS, SCF-treated cells were re-seeded with 1 UI/ml Epo in the presence or absence of 2 µM IM for 24 h. Cells cultured with 1 UI/ml Epo in the presence or absence of IM were used as controls. At indicated times, cells were labeled with 200 ng/ml [^125^I]SCF for 90 min at 4°C and the radioactivity was determined. Results were expressed as percentages of [^125^I]SCF binding to Epo alone at time 0 and are the mean (± SD) of three independent experiments.

We then addressed the question of the reversibility of imatinib effect on c-Kit. We treated UT-7/Epo cells for 2 h with either imatinib or SCF which provoked a 50 or 75% reduction of CD117 expression, respectively. Cells were washed and seeded for an additional period of 22 h in Epo-containing culture medium without imatinib or SCF. CD117 started to be re-expressed 2 h after imatinib or SCF withdraw and reached the baseline after 24 h demonstrating that the effect was reversible (Figure 3A).To investigate the impact of imatinib on the expression of functional receptor at cell surface after ligand-induced down-regulation, UT-7/Epo cells were pre-incubated with 50 ng/ml SCF for 2 h to induce c-Kit down-regulation before adding imatinib to Epo-containing medium for 24 h. While [^125^I]SCF binding was reduced by 75% by a 2-h pre-treatment with SCF, [^125^I]SCF ligation remained low over a period of 24 h in the presence of imatinib (Figure 3B) demonstrating that imatinib prevented c-Kit re-expression at the cell surface. By contrast, expression of functional c-Kit receptors was normalized when cells were re-seeded in Epo-containing medium without imatinib. Notably, a slight increase in c-Kit expression in UT-7/Epo maintained under Epo is observed and maybe correlated to the cell proliferation rate. This suggests that imatinib may inhibit either c-Kit protein synthesis or its cell surface stability.

### Imatinib does not inhibit c-Kit protein neosynthesis and maturation

**Figure 4:**
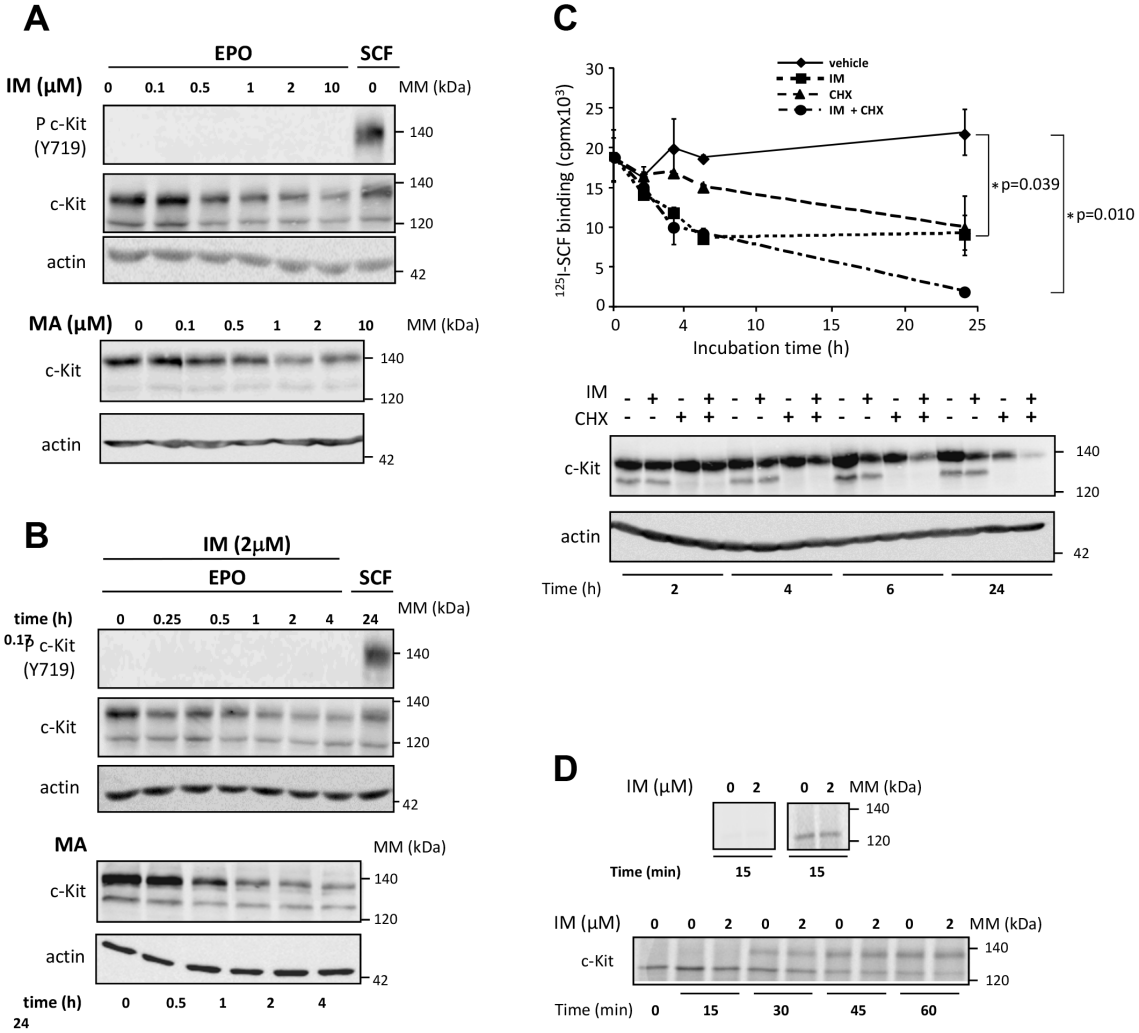
Imatinib or masitinib induces the down regulation of c-Kit but does not influence c-Kit neosynthesis and stability. **A and B. Expression of mature (140 kDa) and immature (120 kDa) forms of c-Kit.** UT-7/Epo cells were incubated with indicated concentrations of imatinib (IM) or masitinib (MA) for 4 h or with 50 ng/ml SCF for 10 min (A) or with 2 µM IM or MA for indicated times (B). Phospho c-Kit (Tyr719) and c-Kit were revealed by immunoblot and actin was used as loading control. All data are representative of at least three independent experiments. **C. Effect of protein synthesis inhibitor cycloheximide and imatinib on the expression of cell surface functional c-Kit. **UT-7/Epo cells were incubated with 2 µM imatinib (IM) or vehicle, in the presence or absence of 500 µM cycloheximide (CHX) for 24 h. At indicated times, cells were labeled with 200 ng/ml [^125^I]SCF for 90 min at 4°C and the radioactivity was determined and expressed as absolute values. Results are the mean of three independent experiments. Alternatively, cell lysates were analyzed for c-Kit expression by immunoblot (lower panel). Actin is used as loading control. **D. c-Kit *de novo* synthesis and maturation.** C-Kit *de novo* synthesis was analyzed in the upper panel UT-7/Epo cells (5.10^5^) were preincubated for 4 h in the presence or absence of 2 µM imatinib (IM) and then labeled with [^35^S]Met/Cys-containing medium for 15 min at 37°C. Immunoprecipitation (IP) was performed using isotypic control (left panel) or anti-c-Kit antibodies (right panel). C-Kit maturation was studied on lower panel. UT-7/Epo (5.10^5^) cells were pulse-labeled with [^35^S]Met/Cys for 15 minutes and chase followed by c-Kit immunoprecipitation using anti-c-Kit antibodies was done for indicated times after incubation with IM (2 µM) or vehicle. Labeled c-Kit was revealed by fluorography. Results are representative of three independent experiments.

Because the maximal effect of TKI on c-Kit expression was delayed compared to the effect of SCF, we first wondered whether imatinib and masitinib affected the expression of mature or immature or both forms of the receptor. We thus analyzed the effect of increasing concentrations or incubation times of the inhibitors on c-Kit protein expression by Western blot using an antibody specific for both forms. As shown in Figure 4A and 4B, both imatinib and masitinib reduced the expression of the mature glycosylated form of 140 kDa from a concentration of 0.5 µM, while the expression of immature 120-kDa form remained constant regardless of the TKI concentration used in UT-7/Epo cells cultured under Epo. The auto-phosphorylation of c-Kit on Tyr719 is only observed after a short term stimulation by SCF (10 min). Consistent with the flow cytometry data, both inhibitors at a concentration of 2 µM efficiently reduced the expression of the mature form of c-Kit after 4 hours of incubation (Figure 4B), as already observed in normal erythroblasts (Figure 1B right).

To investigate whether imatinib modulate c-Kit synthesis and maturation, we first used 500 µM of cycloheximide (CHX) to block protein synthesis. UT-7/Epo cells were incubated with CHX alone or in combination with imatinib at indicated times and functional c-Kit receptors were quantified by measurement of [^125^I]SCF binding. In accordance with a 24-h long half-life of this receptor, CHX alone reduced the binding by 50% after 24 h [11]. While [^125^I]SCF binding decreased by 50% after 6 h of incubation with imatinib only and then remained constant, cell incubation with both CHX and imatinib reduced c-Kit expression by 90% after 24 h (Figure 4C, upper panel). As expected, CHX affected both mature 140 kDa-form and immature 120 kDa form of c-Kit, while imatinib only altered the expression of the mature form (Figure 4C, bottom panel). Finally, imatinib and CHX had an additive inhibitory effect on the expression of mature and functional c-Kit protein (Figure 4C). Altogether, these results suggest that the effect of imatinib may differ from that of CHX suggesting that imatinib did not affect protein synthesis but induced c-Kit degradation.

To precise whether imatinib could modulate c-Kit synthesis, UT-7/Epo cells were pre-incubated for 4 h in the presence or absence of 2 µM imatinib. Then, [^35^S]Cys/Met was incorporated for a pulse of 15 min. Radio-labeled c-Kit was revealed after immunoprecipitation. As shown in Figure 4D (upper panel), the level of immunoprecipitated c-Kit was identical in both conditions suggesting that imatinib did not affect c-Kit *de novo* synthesis. To investigate the effect of imatinib on c-Kit maturation, we labeled UT-7/Epo cells for 15 min with [^35^S]Cys/Met before to chase over a period of 1 h in the presence or absence of 2 µM imatinib. As shown in Figure 4D (bottom panel), imatinib did affect neither the neosynthesis of the 120 kDa form nor the level of 140 kDa mature form of c-Kit confirming that protein neosynthesis and its glycosylation was not impaired by the drug. Taken together, these data show that imatinib did not alter c-Kit biosynthesis. Its additive effect with CHX-mediated inhibition could lead to hypothesize that imatinib may alter c-Kit protein stability.

### Imatinib or masitinib induces c-Kit internalization and its degradation by the lysosomes pathway

**Figure 5:**
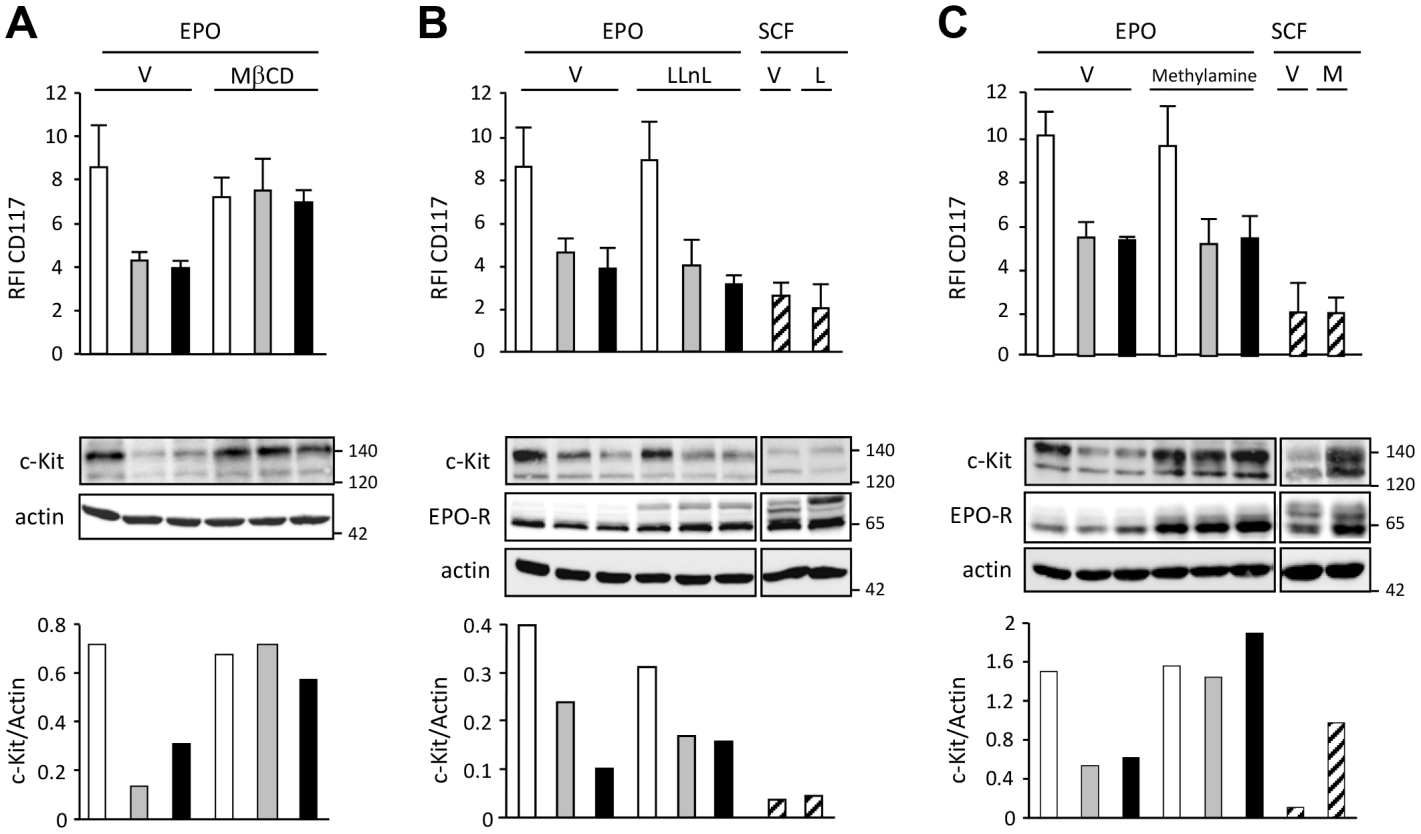
Imatinib or masitinib induces c-Kit internalization and its degradation by lysosome pathway. **A.** For analysis of internalization, UT-7/Epo cells were pre-incubated with 50 µM of methyl-β-cyclodextrine (MβCD) or vehicle (V) for 30 min then treated during 4 hours with imatinib (grey bars) or masitinib (black bars) or Epo alone (white bars). **B.** For the analysis of proteasomal degradation, UT-7/Epo cells were incubated with 1 UI/ml of Epo or 50 ng/ml of SCF (hatched bars), and 50 µM of LLnL (L) or vehicle (V) for 20 min at 37°C before treatment with 2 µM IM (grey bars) or MA (black bars) or Epo alone (white bars) for 4 h. **C.** For the analysis of lysosomal degradation, UT-7/Epo cells were incubated with 1 UI/ml Epo or 50 ng/ml SCF, and 100 µM methylamine (M) or vehicle (V) for 20 min before treatment with 2 µM IM (grey bars) or MA (black bars) or Epo alone (white bars) for 4 h. Expression of c-Kit by flow cytometry (cell surface) and by immunoblot. Actin was used as loading control. Data are representative of three independent experiments and the quantification of one experiment is shown.

To investigate the mechanism of c-Kit modulation by imatinib or masitinib, we first incubated UT-7/Epo cells with 50 µM methyl β cyclodextrin (MbCD) for 30 min before adding 2 µM imatinib or masitinib for 4 h. MβCD has been shown to inhibit internalization of cell surface proteins regardless of their association with raft microdomains [21], [22]. As shown in Figure 5A, MβCD fully blocked the inhibitory effect of TKI on CD117 expression (upper panel) by preserving the expression of mature 140-kDa form of c-Kit under TKI. This suggests that imatinib or masitinib induced the internalization of c-Kit. To precise the pathway of c-Kit degradation following imatinib or masitinib-driven internalization, we pre-incubated the cells with either LLnL, a proteasome inhibitor, or methylamine which could prevent lysosome acidification and inhibits protein degradation by lysosome proteases. We observed that the decrease of CD117 or the disappearance of mature c-Kit was not affected by LLnL (Figure 5B), while the disappearance of the mature 140-kDa form was rescued by methylamine (Figure 5C). Epo-induced EpoR degradation was tested to ascertain the effects of LLnL and methylamine since we have previously demonstrated that proteasome inhibitors protect the cell surface form of the activated EpoR [20]. As expected, LLnL prevented the degradation of the mature form of EpoR (figure 5B). Interestingly, methylamine did not rescue membrane c-Kit expression tested by flow cytometry neither in SCF- nor in TKI-treated cells suggesting that receptors accumulate inside rather than being re-expressed at plasma membrane. We conclude from these experiments that c-Kit was internalized upon SCF binding or imatinib or masitinib treatment in the absence of ligand and degraded through the lysosome pathway.

### c-Cbl is not implicated in imatinib-driven internalization and degradation of c-Kit

**Figure 6:**
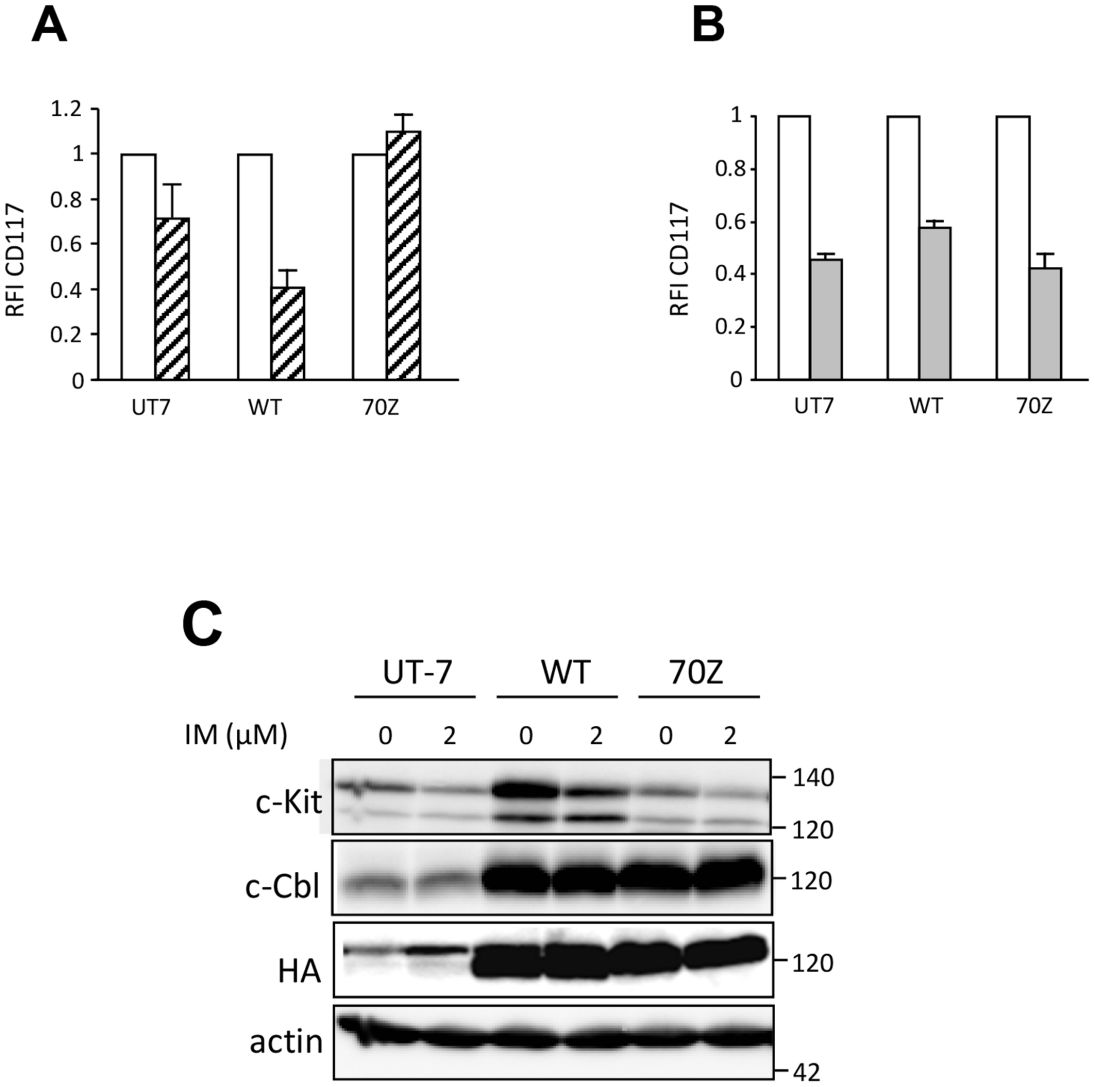
c-Kit internalization occurs independently of c-Cbl. **A. Cell surface expression of c-Kit after SCF binding.** UT-7/Epo either non transfected (UT-7), or transfected with WT c-Cbl (WT) or 70Z c-Cbl mutant (70Z) were cultured with 1 UI/ml Epo (white bars), starved and then stimulated for 5 min with 250 ng/ml SCF (hatched bars). C-Kit cell surface expression was determined by flow cytometry. Results were expressed as RFI of CD117 normalized to cells in Epo alone and are the mean of three independent experiments. **B. Cell surface expression of c-Kit after imatinib treatment.** Parental, WT c-Cbl and 70Z c-Cbl-UT-7/Epo cells cultured with 1 UI/ml Epo (white bars) were treated for 24 h with 2 µM imatinib (grey bars). C-Kit cell surface expression was determined by flow cytometry and the results were expressed as RFI of CD117 normalized to cells in Epo alone and are the mean of three independent experiments. **C. Immunoblot analysis of c-Kit after imatinib treatment.** Parental, *wt*-c-Cbl and 70Z c-Cbl-UT-7/Epo cells were incubated with 2 U/ml Epo in the presence or absence of 2 µM IM for 24 h. Immunoblots using anti-c-Kit, c-Cbl and HA antibodies. Actin is used as loading control. Results are representative of three independent experiments.

It has been previously shown that interaction between c-Kit and E3 ubiquitin ligase c-Cbl proteins, upon SCF stimulation results in their mutual degradation [9], [23]. C-Kit binds to and induces the phosphorylation of c-Cbl proteins, which in turn act as E3 ubiquitin ligases, mediating the ubiquitination, internalization and lysosome degradation of c-Kit. To investigate whether c-Cbl was implicated in the down-regulation of c-Kit by imatinib, we expressed either a mutant 70Z-c-Cbl which is a 17-amino acid deletion mutant (at the boundary of the linker and the RING finger domain), abrogating the E3 ubiquitin ligase function of c-Cbl or the *wt* c-CBL protein in UT-7/Epo cells. Notably, c-Kit expression in UT-7/Epo harboring a wt c-CBL is reproductively increased in comparison to UT-7/Epo parental cells. However, this overexpression of c-Kit is not associated with a high rate of proliferation under SCF stimulation (data not shown) and is actually not investigated. The cell surface expression of c-Kit decreased in response to SCF in the parental UT-7/Epo cell line and in the *wt* c-Cbl-transfected UT-7/Epo cells, while it remained stable in the 70Z-c-Cbl-transfected cells (Figure 6A). We then treated the cells with 2 µM imatinib for 4 h before analyzing CD117 expression by flow cytometry and c-Kit protein by Western blot. As shown in Figures 6B and 6C, 70Z-c-Cbl did not prevent the disappearance of CD117 from plasma membrane or the decrease of mature 140-kDa form induced by the drug. This result shows that c-Cbl was not implicated in the degradation of c-Kit upon imatinib treatment.

### Imatinib does not modulate the content of phosphotyrosine-peptides

Since IM could inhibit several tyrosine kinases in addition of c-Kit, we tested whether IM modified overall tyrosine phosphorylation in UT-7/Epo cells. We performed a global and quantitative analysis of the tyrosine phosphorylated proteins after 4 hours of treatment with IM. To do so, we used the SILAC labeling method [24]. UT-7/Epo cells were cultured for at least 3 weeks with isotopically labeled Lys and Arg. We then verified that more than 95% Lys and Arg residues of each analyzed proteins were isotopically labeled. Heavy amino acids-labeled cells were then treated for 4 h with IM whereas cells labeled with light amino acids received DMSO alone. Proteins from treated and untreated cells were then mixed and digested with trypsin. Tyrosine phosphorylated peptides were immunoprecipitated and analyzed by mass spectrometry (MS). For each identified peptide, ratio of MS signal intensities for heavy to light forms allowed to determine whether or not IM affected the phosphorylation of this peptide. Three independent experiments were performed and peptides identified and quantified in at least two experiments are reported in Table 1. As shown in this table, a single protein (APLP2) showed more than 2 fold change in tyrosine phosphorylation after IM treatment. Thus, IM treatment did not induce a global modification in tyrosine phosphorylation in UT-7/Epo cells. In particular, the phosphorylation of several Src family kinases (Lyn, Hck, Yes) was not modified by IM. Thus, it seems unlikely that IM mediates c-Kit degradation by inducing a global modification of protein phosphorylation on tyrosine residues.

**Table 1:** List of proteins and identified peptides presenting a tyrosine phosphorylation modification after imatinib (IM) treatment in UT-7/Epo cells.

Protein names	Gene names	peptide	Ratio H/L	Position	Range
Amyloid-like protein 2	APLP2	MQNHGYENPTYK	0,47985	Y 755	0,43428–0,52542
Amyloid-like protein 2	APLP2	MQNHGYENPTYK	0,5596	Y 750	0,53448–0,58471
SHC-transforming protein 1	SHC1	EIFDDPSYVNVQNIDK	0,57833	Y 428	0,49184–0,66481
Phosphatidylinositol 3,4,5-trisphosphate 5-phosphatase 1	INPP5D	EKIYDFVK	0,67383	Y 865	0,62630–0,72135
Signal transducer and activator of transcription 5	STAT5	AVDGYVKPQIK	0,71666	Y 694	0,53393–0,81895
Mucin-1	MUC1	YVPPSSTDRSPYEK	0,75444	Y 1238	0,63110–0,87778
Cyclin-dependent kinase 1	CDK1	IGEGTYGVVYKGR	0,77535	Y 19	0,54629–1,00440
Tyrosine-protein kinase Lyn	LYN	SIDNGGYYISPR	0,84216	Y 193	0,81260–0,87172
Tyrosine-protein phosphatase non-receptor type 18	PTPN18	SAEEAPIYSK	0,85268	Y 389	0,83351–0,87185
Catenin delta-1	CTNND1	INGPQDHSHIIYSTIPR	0,86045	Y 96	0,81945–0,90145
Alpha-enolase	ENO1	AAVPSGASTGIYEAIEIR	0,89205	Y 44	0,87500–0,90910
Non-receptor tyrosine-protein kinase TYK2	TYK2	IIAQAEGEPCYIR	0,89579	Y 292	0,87173–0,93281
Schlafen family member 11	SLFN11	NADPIAKYIQK	0,92243	Y 740	0,75395–1,09090
Receptor-type tyrosine-protein phosphatase alpha	PTPRA	VVQEYIDAFSDYANFK	0,94168	Y 809	0,90195–0,98141
Serine/threonine-protein kinase PRP4 homolog	PRPF4B	ICDFGSASHVADNDITPYIVSR	0,94375	Y 849	0,93596–0,95257
Junctional adhesion molecule A	F11R	VIYSQPSAR	0,95872	Y 284	0,80503–1,11240
Glycogen synthase kinase-3	GSK3	GEPNVSYICSR	0,97317	Y 279	0,97190–0,97444
Elongation factor 1-alpha 1	EEF1A1	STTTGHIIYK	0,98889	Y 29	0,97503–1,00510
Tyrosine-protein phosphatase non-receptor type 11	PTPN11	IQNTGDYYDIYGGEK	0,99527	Y 62	0,95104–1,03950
Tyrosine-protein kinase JAK2	JAK2	REVGDYGQIHETEVIIK	0,99782	Y 570	0,95137–1,02940
Mitogen-activated protein kinase 14	MAPK14	HTDDEMTGYVATR	1,00044	Y 182	0,95259–1,04820
Cyclin-dependent kinase 1	CDK1	IGEGTYGVVYK	1,01198	Y 15	0,99314–1,03860
Tyrosine-protein kinase HCK	HCK	VIEDNEYTAR	1,01576	Y 411	0,93732–1,09420
Cyclin-dependent kinase 3	CDK3	IGEGTYGVVYK	1,0163	Y 15	1,00630–1,02170
Tyrosine-protein kinase Lyn	LYN	VIEDNEYTAREGAK	1,02901	Y 397	0,98982–1,06820
Homeodomain-interacting protein kinase 1	HIPK1	AVCSTYIQSR	1,05743	Y 352	1,03746–1,07660
Transgelin-2	TAGLN2	ANRGPAYGISR	1,06637	Y 8	0,98633–1,14640
Dual specificity tyrosine-phosphorylation-regulated kinase 1	DYRK1	IYQYIQSR	1,14687	Y 321	1,06570–1,27760
Mitogen-activated protein kinase 3	MAPK3	IADPEHDHTGFITEYVATR	1,14715	Y 204	1,11940–1,17490
Mitogen-activated protein kinase 1	MAPK1	VADPDHDHTGFITEYVATR	1,20773	Y 187	1,01550–1,39270
Tyrosine-protein kinase Yes	YES1	IIEDNEYTAR	1,2854	Y 426	1,15650–1,44170

A ratio H(eavy)/L(ight)<0.5 is associated with a significant decrease in tyrosine phosphorylation of the indicated Y residue after IM treatment. At the opposite, a ratio H/L>2 is associated with a significant increase in tyrosine phosphorylation of the indicated Y residue after IM treatment. All the peptides were at least identified in two different experiments. For each peptide, the range of ratio H/L represent the dispersion observed between the experiments.

### The integrity of c-Kit ATP pocket is crucial for imatinib-induced internalization

We suspected that internalization was driven by direct interaction between imatinib and the ATP binding pocket of c-Kit. To test this hypothesis, we used a mutated form of c-Kit, the so called “gate keeper” mutant, which contains a T670I substitution within the ATP pocket. This mutation abolishes the sensitivity to imatinib by inducing a substantial deformation of the ATP pocket which prevents the binding of imatinib, while the multikinase inhibitor sorafenib retains a better access to the ATP pocket and an inhibitory activity to this mutant. [25], [26], [27].

**Figure 7:**
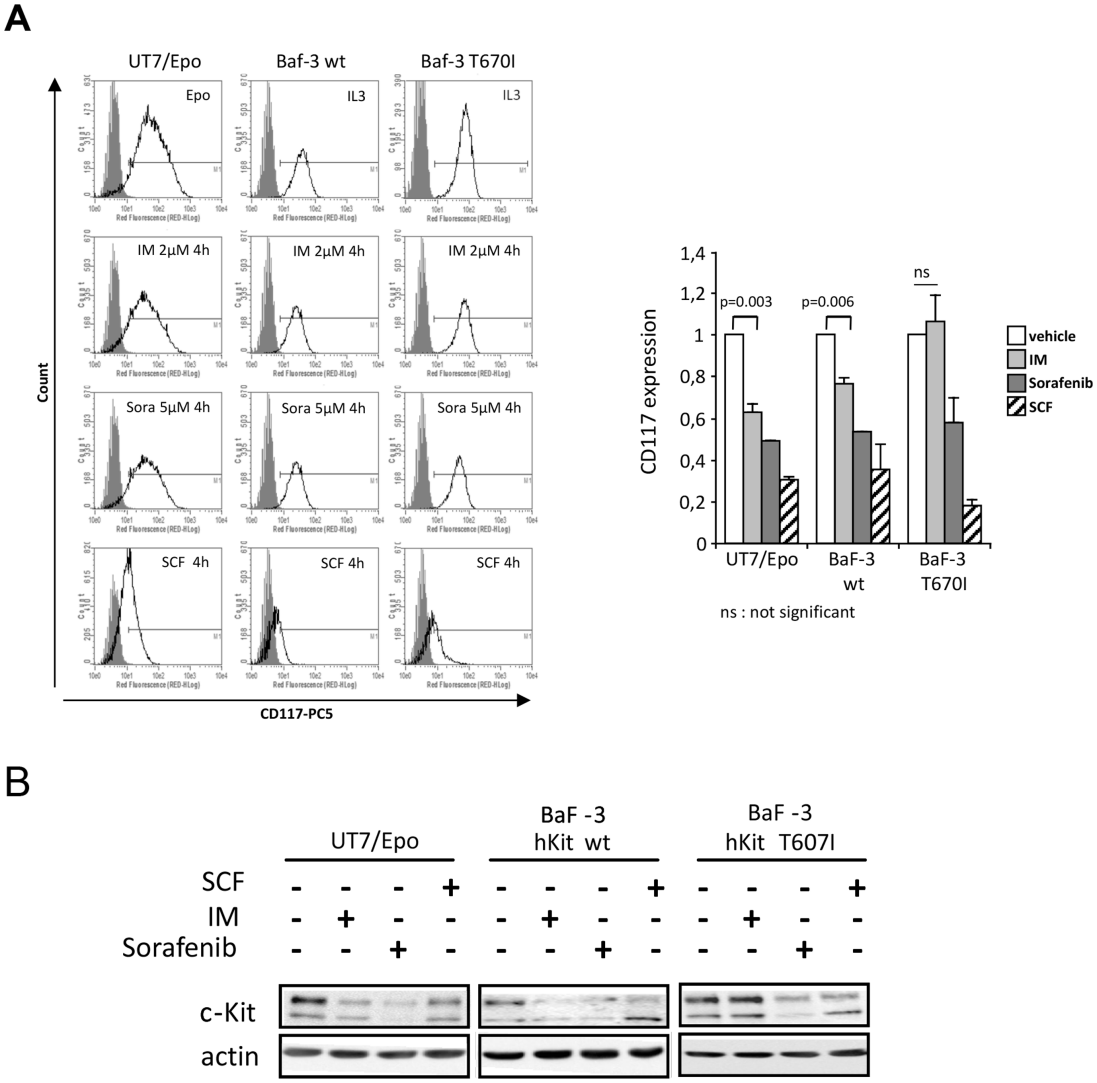
The integrity of c-Kit ATP pocket is crucial for imatinib-induced internalization. UT-7/Epo, BaF-3 hKit WT and BaF-3 hKit T670I cultivated with Epo (1 UI/ml) or IL3 (1 ng/ml) were incubated 4 h with 2 µM Imatinib or 5 µM sorafenib or vehicle or stimulated 10 minutes with 50 ng/mL SCF. **A**. Cell surface expression of c-Kit by flow cytometry using CD117 antibody (black line) compared to isotypic antibody (grey line). Results were expressed as RFI of CD117 normalized to cells in control conditions. The results are the mean of three independent experiments. **B**. Western Blot analysis of cell lysates. Actin is used as loading control. Results are representative of three independent experiments.

The effects of imatinib on c-Kit expression were analyzed in the murine BaF-3 cell line transfected with human T670I c-Kit mutant or *wt*-c-Kit. As shown in Figure 7A, internalization of Kit by imatinib was detected in UT-7/Epo and *wt*-c-Kit BaF3 cells, but not in the T670I c-Kit mutant BaF-3 cells. Sorafenib used at 5 µM was able to induce c-Kit internalization in the two cell types. In western blot experiments, we observed that IM was unable to decrease the expression of the mature form of T670I c-Kit mutant, while the *wt* form of the receptor decreased in UT-7/Epo or in *wt*-c-Kit BaF3 cells (Figure 7B). As expected, the expression of the mature form of T670I c-Kit mutant was down regulated in the presence of sorafenib. These data indicate that the accessibility of the ATP pocket is crucial for imatinib-induced c-Kit internalization process.

## Discussion

In the present work, we have demonstrated that imatinib induces the down-regulation of the mature and functional form of c-Kit in the absence of SCF. The mechanism of TKI-induced c-kit down-regulation implicates both internalization and lysosomal degradation of the receptor. This effect is observed as long as the cells are exposed to the drugs and is reversible after TKI withdrawal. Our data suggests that the integrity and occupancy of the ATP pocket by TKI is required for initiation of c-Kit down-regulation.

Our main observation is that c-Kit interacting with imatinib in the absence of ligand could be processed for internalization. This is consistent with the capacity of mutant kinase dead receptors to internalize. The kinetics of internalization upon SCF ligation or imatinib were different. While activated receptor upon ligand binding is rapidly internalized, the internalization of c-Kit by imatinib is slow suggesting that the mechanism could be quite different. Activation of the E3 ubiquitin ligase c-Cbl, is a key step for EGF-R or c-Kit endocytosis after ligand binding or active mutant c-Kit endocytosis, and their subsequent targeting to the lysosomal pathway after mono-ubiquitination [9], [23], [28]. The recruitment of c-Cbl to c-Kit requires the phosphorylation of tyrosine residues located close to the transmembrane domain (Y568 and Y760) and in the C-terminus part of the receptor (Y936) [9], [28]. These tyrosine residues are phosphorylated by the receptor tyrosine kinase upon ligand binding, and subsequent receptor internalization is achieved when c-Cbl becomes phosphorylated by Src kinase family members [4], [29], [30], [31]. Here, we found that c-Kit internalization induced by imatinib occurs while c-Kit kinase activity is abrogated, which may prevent the activation of c-Cbl and consequently delay the process.

Because the spectrum of TKI targets includes both membrane receptors like c-Kit and several intracellular kinases, we should ask whether the internalization of c-Kit is a side effect due to the inhibition of other intracellular kinases. To explore this hypothesis, we have analyzed the phosphotyrosine peptides in the UT-7/Epo cell line cultured with Epo before and after induction of c-Kit internalization with imatinib. We did not observe significant modifications of phosphotyrosine peptide content, including those corresponding to major signaling pathways downstream Epo receptor including the. STAT5 and ERK1/2 pathways. This is consistent with the fact that IM did not modify Epo-induced UT-7/Epo cell proliferation (Figure 1D). This demonstrates that imatinib does not induce a significant modification of the activity of most tyrosine kinases in these cells.

Our data support the idea that internalization could be initiated after the binding of imatinib to the ATP binding pocket. We used the T670I mutant c-Kit unable to bind imatinib and responsible for myeloproliferative disorder in mice and imatinib-resistant GIST in humans [26], [32]. Indeed, up to 10 µM imatinib was ineffective to block T670I mutant –driven cell proliferation, while kinase inhibitor sorafenib, which inhibits wt c-Kit at a concentration of 5 µM efficiently reduced this proliferation [27,33 and our data not shown]. Sorafenib also efficiently improved response rate and overall survival in patients with metastatic renal cell carcinoma overexpressing *wt* c-Kit [34]. In our work, we show that both sorafenib and imatinib induced the down-regulation of *wt* c-Kit suggesting that internalization consecutive to targeting of the ATP binding pocket is a common mechanism for various TKI. By contrast, sorafenib but not imatinib could induce the internalization of c-Kit when mutated at T670 which exhibits a deformation of the ATP binding pocket previously shown to prevent the binding of imatinib. We conclude from these experiments that binding in the ATP pocket of c-kit is a prerequisite for subsequent initiation of the internalization process by TKI.

The remaining question is the consequence of imatinib binding on the overall conformation of the receptor. In 2004, Mol and colleagues [35] have shown that c-Kit, in the absence of ligand, may exist in two different pre-activated and auto-inhibited conformations. To further describe the auto-inhibited conformation, they generated a series of point mutations, and demonstrated the importance of juxta-membrane domain for the stabilization of the protein. They also demonstrated that IM could disrupt the conformation thus abrogating the auto-inhibited state. We suspect that when released from auto-inhibition, the receptor should initiate its internalization process. This was confirmed by X-ray crystallography of c-Kit-imatinib complex showing that imatinib binds to a conformational inactive monomeric form of c-Kit. We suggest that this modification could expose an internalization specific motif that is normally buried in inactive receptors. However, since internalization kinetics after TKI treatment is much slower than after SCF stimulation, it is likely that efficiency of this process is poor compared to the down-regulation mechanism involving c-Cbl after SCF stimulation.

Finally, imatinib-dependent c-Kit down-regulation drastically impacts on the rate of erythroblast maturation *in vitro*. Thus, c-Kit expression alone seems to be sufficient to delay the terminal differentiation of erythroid cells in the absence of SCF. Whether c-Kit could behave as a molecular adaptor in this process or is activated to low levels by a mechanism that remains to be determined deserves supplementary investigations.

In conclusion, our results describe the mechanism of long-term induction of c-Kit internalization by TKI. Prolonged down-regulation and degradation of c-Kit by imatinib may add to the rapid inhibitory effect of the drug on c-Kit tyrosine kinase activity in tumors in which overexpression of wt-c-Kit worsens the prognosis.

## Supporting Information

Figure S1 **Imatinib or masitinib decreases cell surface expression of mature and functional c-Kit in BaF3 hKit WT and TF-1 cells. A and B.** Cell surface expression of c-Kit. Murine BaF3 were stably transfected with a human c-Kit WT and incubated with indicated concentrations of imatinib (IM) (grey bars) or masitinib (MA) (black bars) for 4 h (A). TF-1 erythroid cell line was incubated with 2 µM of imatinib (IM) (grey bars) or 2 µM of masitinib (MA) (black bars) for 4 h (B). C-Kit cell surface expression was determined by flow cytometry. Results were expressed in Ratio of median Fluorescence Intensity (RFI), and the values were normalized to untreated cells. Results are the mean of at least three independent experiments.
(TIF)
