# Supplementary figures and images for: Tyrosine Kinase Inhibitors Induce Down-Regulation of c-Kit by Targeting the ATP Pocket
Source: PLoS One. 2013 Apr 23;8(4):e60961. doi: 10.1371/journal.pone.0060961 (PMC3634048; doi:10.1371/journal.pone.0060961)

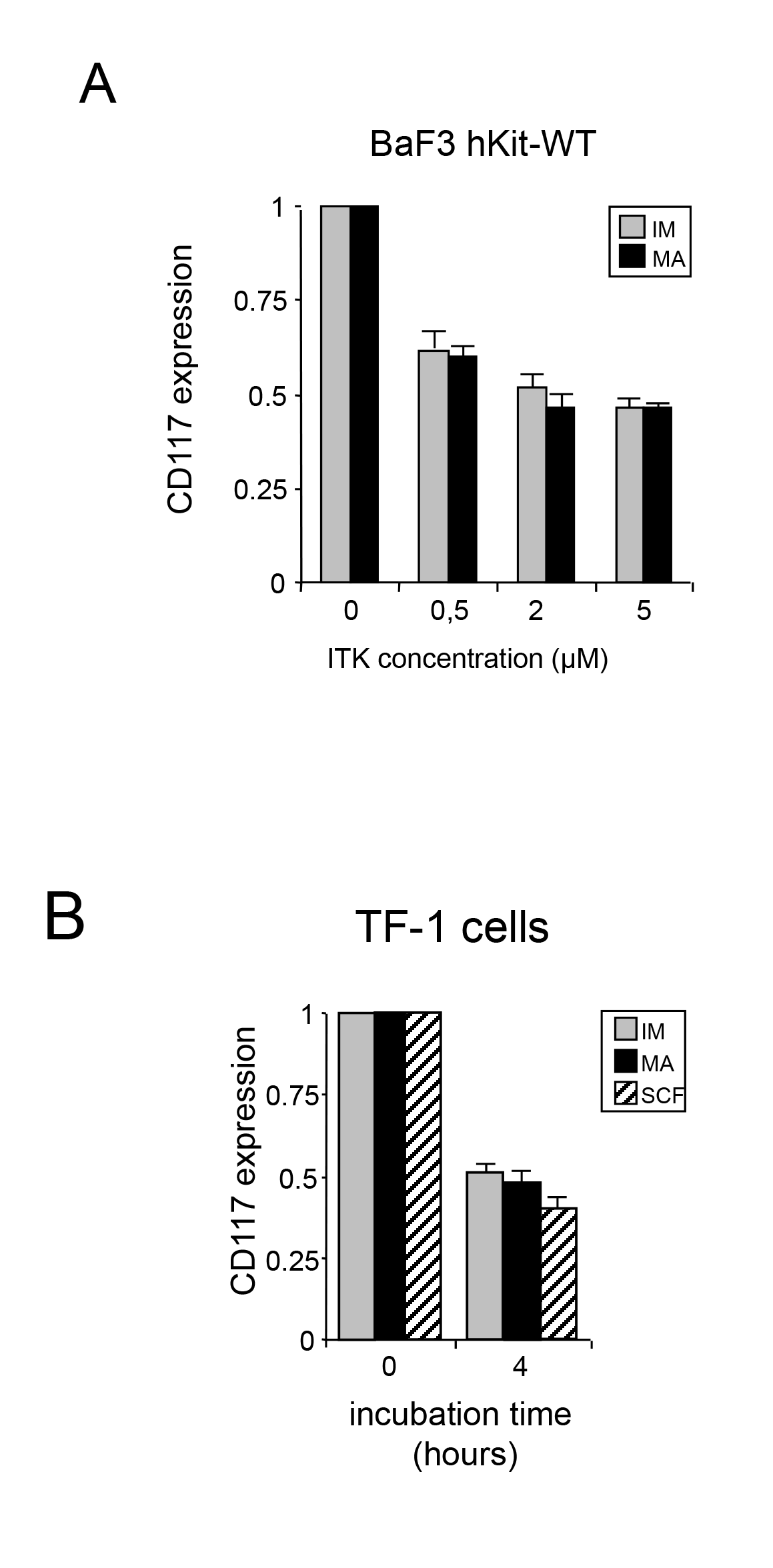

Supplement: Figure S1 — Imatinib or masitinib decreases cell surface expression of mature and functional c-Kit in BaF3 hKit WT and TF-1 cells. A and B. Cell surface expression of c-Kit. Murine BaF3 were stably transfected with a human c-Kit WT and incubated with indicated concentrations of imatinib (IM) (grey bars) or masitinib (MA) (black bars) for 4 h (A). TF-1 erythroid cell line was incubated with 2 µM of imatinib (IM) (grey bars) or 2 µM of masitinib (MA) (black bars) for 4 h (B). C-Kit cell surface expression was determined by flow cytometry. Results were expressed in Ratio of median Fluorescence Intensity (RFI), and the values were normalized to untreated cells. Results are the mean of at least three independent experiments. (TIF) [file pone.0060961.s001.tif]
